# Mugen-UMAP: UMAP visualization and clustering of mutated genes in single-cell DNA sequencing data

**DOI:** 10.1186/s12859-024-05928-x

**Published:** 2024-09-27

**Authors:** Teng Li, Yiran Zou, Xianghan Li, Thomas K. F. Wong, Allen G. Rodrigo

**Affiliations:** 1 School of Biological Sciences, University of Auckland, Auckland, New Zealand; 2 Research School of Biology, Australian National University, Canberra, ACT Australia; 3 School of Computing, Australian National University, Canberra, ACT Australia

**Keywords:** UMAP, Visualization, Clustering, Single-cell DNA sequencing, Gene mutation

## Abstract

**Background:**

The application of Uniform Manifold Approximation and Projection (UMAP) for dimensionality reduction and visualization has revolutionized the analysis of single-cell RNA expression and population genetics. However, its potential in single-cell DNA sequencing data analysis, particularly for visualizing gene mutation information, has not been fully explored.

**Results:**

We introduce Mugen-UMAP, a novel Python-based program that extends UMAP’s utility to single-cell DNA sequencing data. This innovative tool provides a comprehensive pipeline for processing gene annotation files of single-cell somatic single-nucleotide variants and metadata to the visualization of UMAP projections for identifying clusters, along with various statistical analyses. Employing Mugen-UMAP, we analyzed whole-exome sequencing data from 365 single-cell samples across 12 non-small cell lung cancer (NSCLC) patients, revealing distinct clusters associated with histological subtypes of NSCLC. Moreover, to demonstrate the general utility of Mugen-UMAP, we applied the program to 9 additional single-cell WES datasets from various cancer types, uncovering interesting patterns of cell clusters that warrant further investigation. In summary, Mugen-UMAP provides a quick and effective visualization method to uncover cell cluster patterns based on the gene mutation information from single-cell DNA sequencing data.

**Conclusions:**

The application of Mugen-UMAP demonstrates its capacity to provide valuable insights into the visualization and interpretation of single-cell DNA sequencing data. Mugen-UMAP can be found at https://github.com/tengchn/Mugen-UMAP

**Supplementary Information:**

The online version contains supplementary material available at 10.1186/s12859-024-05928-x.

## Background 

Uniform manifold approximation and projection (UMAP) [1] has been widely used for visualization and nonlinear dimensionality reduction in single-cell RNA expression datasets [2], and has also been utilized in population genetics to study population structure [3]. However, the application of UMAP in single-cell DNA data analysis remains notably limited. Here, we developed a new program named Mugen-UMAP to apply UMAP innovatively to single-cell DNA sequencing data for the analysis and visualization of gene mutation information (e.g., in single-cell somatic mutations). Furthermore, we demonstrate the application of UMAP algorithm [1] to analyze single-cell whole-exome sequencing (WES) data from 12 non-small cell lung cancer (NSCLC) patients [4], using gene mutation information from detected somatic mutations, revealing distinct cell clusters corresponding to the various histological subtypes of NSCLC. We also applied Mugen-UMAP to the additional 9 single-cell WES datasets across six different cancer types, uncovering interesting cluster patterns that may merit further exploration. This approach provides valuable insights into the identification of clusters and interpretation of single-cell DNA sequencing data.

## Materials and methods

### Implementation

**Fig. 1:**
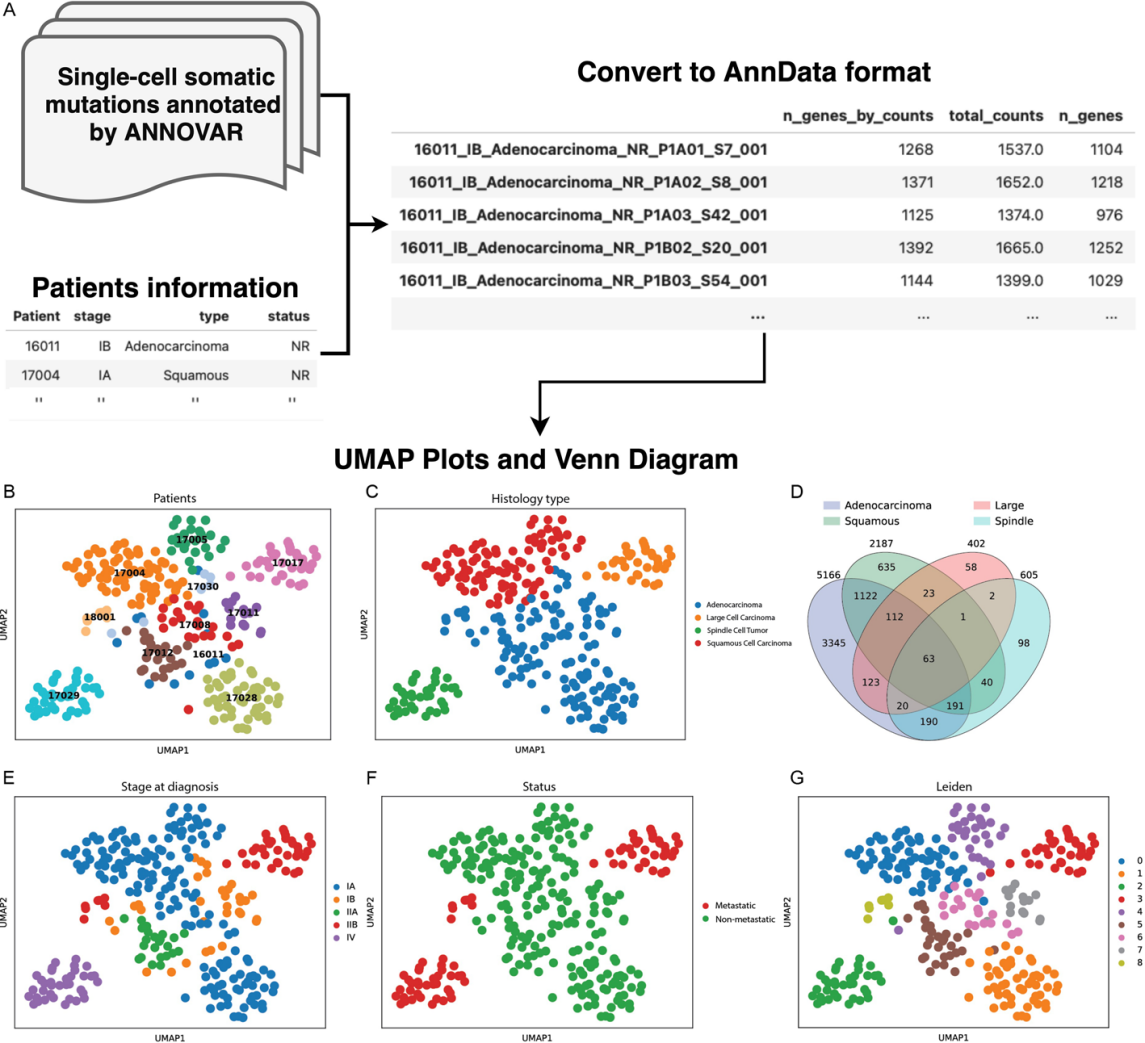
The diagram of Mugen-UMAP workflow. **A** Single-cell somatic mutations annotated by ANNOVAR, coupled with corresponding patient information, were converted into the AnnData format. Subsequently, UMAP projections colored according to (**B**) Patient ID, **C** histology type, **E** diagnostic stage, **F** metastatic status, **G** Leiden algorithm, and **D** the Venn diagram were generated, along with various statistical analyses, utilizing the single-cell DNA sequencing data. The numbers in the Venn diagram represent the counts of mutated genes shared among the different histological subtypes of NSCLC, including adenocarcinoma, squamous cell carcinoma, large cell carcinoma, and spindle cell carcinoma

Mugen-UMAP is implemented in Python with three main features (Fig. 1). (i) *convert*, allows users to convert their somatic single-nucleotide variants (SNVs) annotation files and the metadata file into AnnData format [5], which stores a data matrix of genes by cells. Each entry in the matrix represents the number of mutations per gene for each cell. The input can be either a ZIP file or a directory containing the annotated mutation files of each cell, generated by ANNOVAR [6] through the annotation of related mutations in the Variant Call Format (VCF). The metadata file should contain the patient ID or sample ID in the first column, along with other related information, such as the type (histology type), stage (diagnostic stage), and relevant numerical data (e.g., number of cells). Our program will automatically select the non-numerical columns for subsequent plotting steps. (ii) *umap*, allows users to plot UMAP projections (e.g., for clinical subjects, colored by Patient ID, histology type, or diagnostic stage) by integrating and adjusting the common workflow of Scanpy [7] (includes (1) removing genes that are mutated in less than 3 cells, (2) excluding cells with less than 30 mutated genes, (3) excluding outlier cells with mutated gene counts that exceed 98% of all samples, (4) normalizing counts in each cell followed by logarithmization, (5) selecting the top 3000 highly variable genes, and regressing out the effects of total counts per cell), and to generate Venn diagram using Venny4Py (https://github.com/timyerg/venny4py), coupled with various summary reports. Moreover, visualizations for each filtering step (along with the corresponding cutoff values) will be generated (e.g., Fig. S1 for the NSCLC dataset), which allow users to assess the impact of the filtering steps and facilitate the optimization of filtering parameters specific to their studies. Furthermore, two clustering algorithms, Leiden [8] and Louvain [9], were provided for detecting cell clusters or patterns. (iii) *all*, execute the full pipeline, including both the *convert* and *umap* functions in sequence.

### Application of Mugen-UMAP to example datasets

To demonstrate the capabilities of Mugen-UMAP, we applied it to a dataset comprising 365 single-cell samples isolated from the primary tumors of 12 NSCLC patients (with a median of 23 cells per patient, ranging from 7 to 71), coupled with one corresponding normal bulk tissue for each patient [4] (Table 1). Whole exome sequencing was performed for all samples using the Illumina platform, achieving an average coverage depth of 198.1X for normal bulk tissues (median depth of 163.8X) and 101.5X for tumor single cells (median depth of 100.1X). Somatic SNVs were detected individually for each tumor single cell sample against the matched normal bulk sample by VarScan v2.4.3 [10], with the default parameters except increasing the minimum read coverage to at least 10 reads in both tumor and matched normal samples. Then, somatic SNVs located within the repeat region (as annotated by RepeatMasker) on the UCSC Table Genome Browser [11] and those falling outside the exon target regions were excluded. To avoid potential low-quality somatic SNV calling, SNVs were retained if these sites could be genotyped by GATK HaplotypeCaller [12] in at least 70% of all samples for each patient.

**Table 1:** 12 non-small cell lung cancer (NSCLC) patients information

Patient ID	Age at Dx	Stage at Dx	Histology	Status^a^	Tumor cells	Filtered cells	Normal Bulk	Mean non-synonymous SNVs^b^	Mutated genes^c^
16,011	40–49	IB	AC	NM for 45 months	18	15	1	1595.9	3047
16,031	70–79	I	SCC	NM for > 3 years*	14	4	1	1986.3	NA
17,004	70–79	IA	SCC	NM for 40 months	71	66	1	194.0	1247
17,005	70–79	IA	SCC	NM for 32 months	27	24	1	374.2	1244
17,008	50–59	IA	AC	NM for 18 months	23	21	1	661.0	2030
17,011	60–69	IB	AC	NM for 14 months	20	14	1	271.8	659
17,012	80–89	IIA	AC	NM for 15 months	23	23	1	306.4	1166
17,017	60–69	IIB	LCC	Metastasis to Lymph nodes, Now deceased	46	33	1	82.7	402
17,028	70–79	IA	AC	NM for 27 months	59	48	1	97.2	586
17,029	60–69	IV	SpCC	Metastasis to Spine, Now deceased	47	36	1	152.6	605
17,030	60–69	IB	AC	NM for 36 months	7	6	1	563.7	589
18,001	70–79	IIB	AC	Metastasis to Lymph nodes	10	7	1	155.7	183

Information for these patients, previously described in Li et al. (2021), has been updated with additional information from single-cell somatic mutation analysis

*Dx* diagnosis, *M* male, *F* female, *AC* adenocarcinoma, *SCC* squamous cell carcinoma, *LCC* large cell carcinoma, *SpCC* spindle cell carcinoma, *NM* non-metastasis

*Patient 16,031 had a wedge resection with completion lobectomy for a positive suture line tumor; the patient is now non-recurrent for more than 3 years following the resection of the residual tumor

^a^Recurrence-free duration for the non-metastasis NSCLC patients are as of their last clinic visit

^b^The mean non−synonymous SNVs have been filtered to exclude genes mutated in less than 3 cells, as well as cells with less than 30 mutated genes

^c^The number of mutated genes that passed all filtering steps, retaining only those genes mutated in at least two cells per patient, were subsequently used to generate the Venn diagram

Furthermore, to showcase the broad applicability of Mugen-UMAP, we obtained 9 single-cell WES datasets from various studies [13–18] (Table 2), encompassing 332 single-cell samples from six different cancer types (including bladder, blood, breast, colon, kidney, and lung). Each dataset represents an individual patient, except for Wu-CRC0827 and WuCRC0827-polyps, which are from the same patient. The pipeline for processing SNV calling of these 9 datasets was described in Borgsmüller et al. [19]. For both example datasets, the mutations in the VCF files of each cell were then annotated using ANNOVAR [6] with the Catalogue of Somatic Mutations in Cancer (COSMIC) database [20], and only non-synonymous SNVs were retained for subsequent analysis. However, for the 9 additional single-cell WES datasets, because the total number of mutated genes remaining after filtering was only 1002, we retained all of these genes for subsequent analysis.

**Table 2:** 9 published single-cell whole-exome sequencing (WES) cancer datasets

Dataset^a^	Tissue	Cells	Filtered cells	SNVs	Mean non-synonymous SNVs^b^	Mutated genes^c^
Li [13]	bladder	55	54	885	37.5	73
Hou [14]	blood	82	71	1387	133.9	249
Wang-ER + [15]	breast	47	46	355	38.8	66
Wang-TNBC [15]	breast	16	15	1472	215.3	312
Wu-CRC0827 [16]	colon	50	50	652	58.2	116
WuCRC0827-polyps [16]	colon	19	19	379	NA	NA
Wu-CRC0907 [16]	colon	50	49	574	46	96
Xu [17]	kidney	20	20	747	56.3	90
Ni [18]	lung	8	8	340	NA	NA

^a^Each of these 9 single-cell WES datasets represents individual patients, with the exception of Wu-CRC0827 and WuCRC0827-polyps, which are from the same patient. The WuCRC0827-polyps dataset corresponds to the colorectal adenomatous polyps from the same patient as the Wu-CRC0827 dataset

^b,c^The annotation is the same as for Table 1

## Results and discussion

**Fig. 2:**
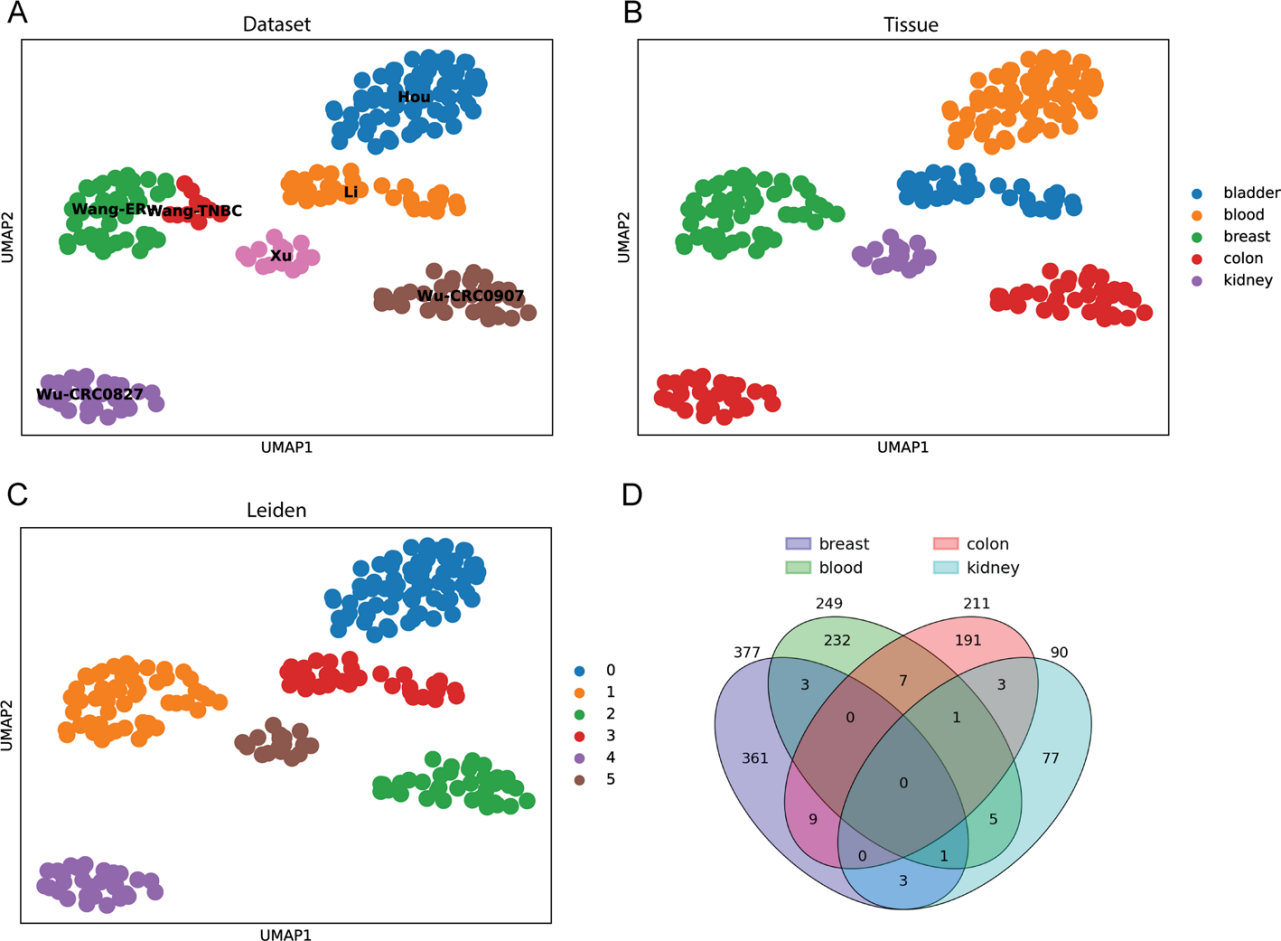
UMAP visualization and Venn diagram of 9 additional cancer datasets. UMAP projections colored according to (**A**) single-cell dataset, **B** cancer tissue, and **C** Leiden algorithm, coupled with the (**D**) Venn diagram showing the number of overlapping mutated genes among different cancer types (breast, blood, colon, and kidney)

We employ the *all* function in Mugen-UMAP, inputting these annotation files and patient information metadata (Tables 1, 2), with the default value to plot UMAP projections for visualizing and identifying cell clusters. Additionally, the Venn diagram was generated to visualize the shared and unique mutated genes among four different groups of patients (Figs. 1, 2).

For the dataset comprising 12 NSCLC patients (Patient-16031 was excluded because no cells passed our filtering criteria) (Table 1), our results revealed four distinct cell clusters corresponding to the different histological subtypes of adenocarcinoma, squamous cell carcinoma, large cell carcinoma and spindle cell carcinoma (Fig. 1C), rather than being based on patient ID, diagnostic stages, or survival status (Fig. 1B, E, F). Moreover, among these groups, adenocarcinoma and squamous cell carcinoma appeared to be more closely located in UMAP space compared to the other two groups. This observation was further supported by the evidence that these two groups shared a higher number of mutated genes with each other than with the large cell and spindle cell carcinomas (Fig. 1D). These results suggest that gene mutations may be associated with the histological subtypes of NSCLC, as evidenced by certain gene mutations that are considered pathognomonic for specific histological subtypes [21]. For example, alterations in *EGFR*, *KRAS*, *SMARCA4*, *STK11*, and *KEAP1* are almost exclusively detected in adenocarcinoma [21], which were also evident in our study (see Supplementary file 3). However, our results may potentially be influenced by the limited sampling, with only one patient each representing large cell carcinoma and spindle cell carcinoma, which could be attributed to the relative rarity of these two subtypes in NSCLC. Additionally, both the Leiden and Louvain algorithms displayed similar clustering patterns, identifying 9 and 7 cell clusters, respectively (Fig. 1G, S2), which partially support our observations that the clusters are associated with the histological subtypes of NSCLC.

For the additional 9 single-cell WES cancer datasets (two of which were excluded because no cells passed our filtering criteria) (Table 2), the results demonstrated six distinct cell clusters in a non-overlapping fashion based on the Leiden algorithm [8] (Fig. 2C). By color-coding the cancer tissues on the UMAP, these clusters separated according to different cancer types, with the exception of the datasets Wu-CRC0827 and Wu-CRC0907, both from colon cancer, which exhibited spatial separation and thus may warrant further investigation to explain why such separation is apparent (Fig. 2B). This observation was further supported by the Venn diagram (Fig. 2D), which indicated that there are no overlapping mutated genes shared among the cancer types of blood, breast, colon, and kidney, with most mutated genes being unique to their respective cancer types.

## Conclusions

Mugen-UMAP, a Python package, extends the application of UMAP to single-cell DNA sequencing data, focusing on the visualization and identification of cell clusters based on gene mutation information. By applying this tool to two different example single-cell WES datasets—one comprising 12 NSCLC patients and another from 8 patients with various cancer types—Mugen-UMAP revealed distinct cell clusters corresponding to different histological subtypes and cancer types, respectively. This pioneering application of UMAP in single-cell WES data analysis offers a new way for visualization, clustering, and interpretation of single-cell DNA sequencing data. In conclusion, Mugen-UMAP is a useful tool for applying UMAP to enhance the analysis and visualization of gene mutation information in single-cell DNA sequencing data.

## Supplementary Information

Supplementary Figure 1. Visualizations of the Mugen-UMAP filtering steps in the NSCLC dataset. **A** Distribution of mutated cells per gene, with a cutoff line indicating that genes mutated in less than 3 cells will be removed. **B** Distribution of mutated genes per cell, showing the lower cutoff for excluding cells with less than 30 mutated genes and the upper cutoff for excluding cells with mutated gene counts exceeding 98% of all samples. **C** Dispersion of highly variable genes, with the black dots representing the top 3000 highly variable genes selected for subsequent analysis.

Supplementary Figure 2. UMAP projections of the Louvain clustering algorithm applied to the NSCLC dataset.

Supplementary file 3. AnnData format of the 12 NSCLC patients dataset.

## Data Availability

The datasets generated and/or analysed during the current study are available in the GitHub repository, https://github.com/tengchn/Mugen-UMAP/tree/main/Examples. Project name: Mugen-UMAP. Project home page: https://github.com/tengchn/Mugen-UMAP. Operating system(s): Platform independent. Programming language: Python. Other requirements: Python3, scanpy, numpy, pandas, venny4py, matplotlib, leidenalg, louvain. License: MIT. Any restrictions to use by non-academics: None.

## Abbreviations

UMAP: Uniform manifold approximation and projection
WES: Whole-exome sequencing
NSCLC: Non-small cell lung cancer
SNV: Single-nucleotide variant
COSMIC: Catalogue of somatic mutations in cancer
