# Supplementary figures and images for: Mugen-UMAP: UMAP visualization and clustering of mutated genes in single-cell DNA sequencing data
Source: BMC Bioinformatics. 2024 Sep 27;25:308. doi: 10.1186/s12859-024-05928-x (PMC11437917; doi:10.1186/s12859-024-05928-x)

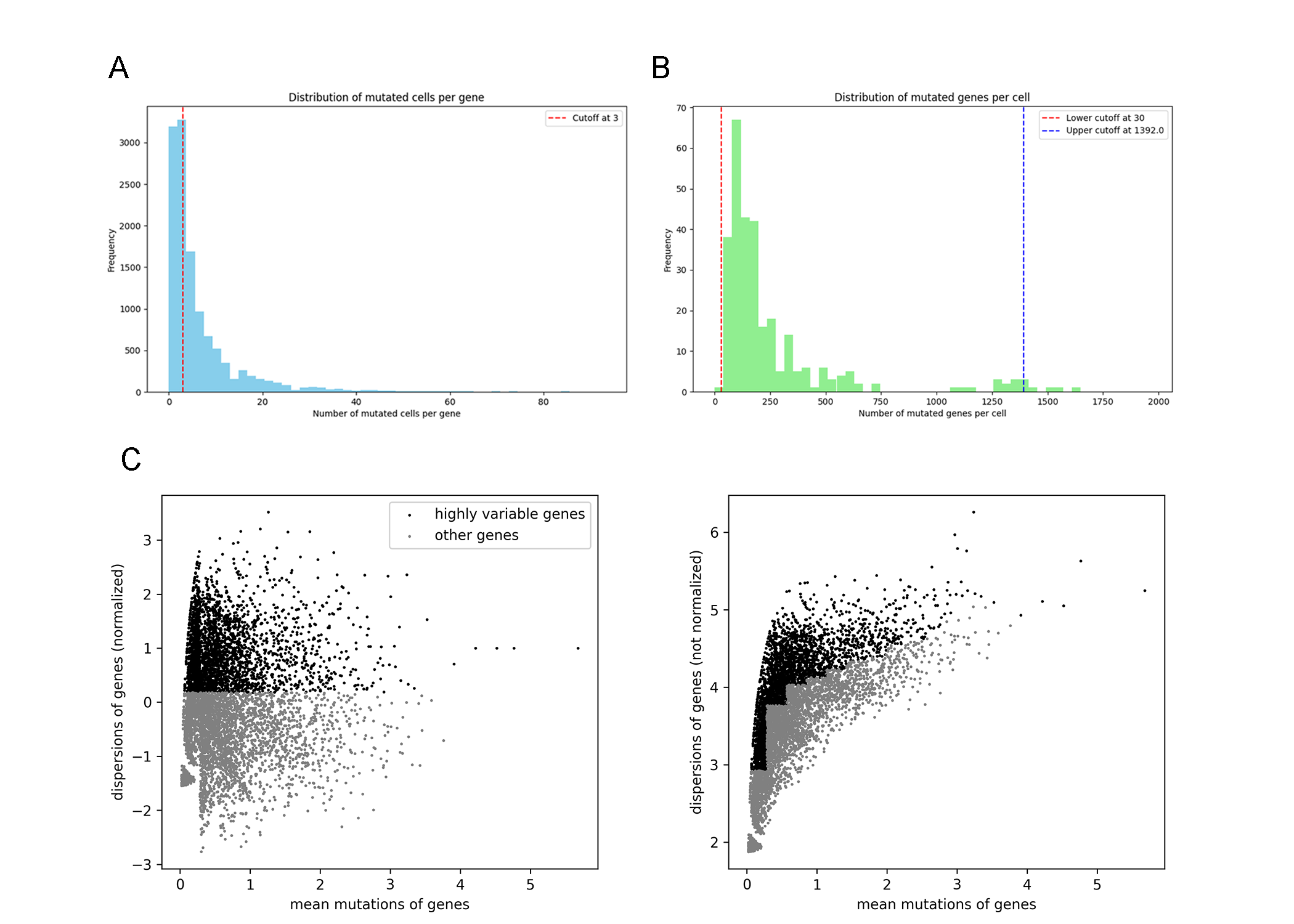

Supplement: Supplementary file 1 — Supplementary Figure 1. Visualizations of the Mugen-UMAP filtering steps in the NSCLC dataset. A Distribution of mutated cells per gene, with a cutoff line indicating that genes mutated in less than 3 cells will be removed. B Distribution of mutated genes per cell, showing the lower cutoff for excluding cells with less than 30 mutated genes and the upper cutoff for excluding cells with mutated gene counts exceeding 98% of all samples. C Dispersion of highly variable genes, with the black dots representing the top 3000 highly variable genes selected for subsequent analysis. [file 12859_2024_5928_MOESM1_ESM.tif]

louvain

UMAP2

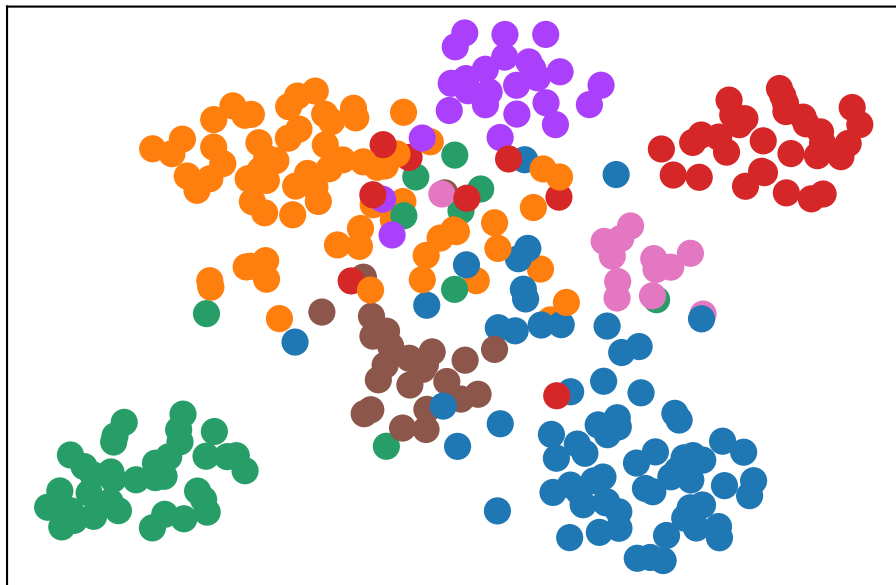

UMAP1

Supplement: Supplementary file 2 — Supplementary Figure 2. UMAP projections of the Louvain clustering algorithm applied to the NSCLC dataset. [file 12859_2024_5928_MOESM2_ESM.pdf]
